# Changes in Peripheral Blood Neutrophils, Lymphocytes and IL-10 in Children with Kawasaki Disease from Different Age Groups Undergoing Intravenous Immunoglobulin: A Retrospective Study

**DOI:** 10.1155/2020/5213451

**Published:** 2020-11-18

**Authors:** Chun Zhang, Xuan Zhang, Jia Shen, Xiaotong Lu, Jian Zhang, Sun Chen

**Affiliations:** ^1^Department of Pharmacy, Xinhua Hospital affiliated to Shanghai Jiao Tong University School of Medicine, Shanghai 200092, China; ^2^Department of Pediatric cardiology, Xinhua Hospital Affiliated to Shanghai Jiao Tong University School of Medicine, Shanghai 200092, China; ^3^Shanghai Jiahui International Health, Shanghai 200233, China

## Abstract

Immunoglobulin intravenous (IVIG) is widely used in mucocutaneous lymph node syndrome, known as Kawasaki disease (KD). However, the patients' inflammatory response during usage remains unclear. In the present study, the association between inflammatory response and lymphocyte count in children with KD from different ages was evaluated before and after IVIG. The medical records of 50 children with KD were retrospectively reviewed and divided into five groups according to age. As compared with the data from healthy children, the relative neutrophil count of all children with KD was increased, and that of lymphocytes was decreased. The neutrophil/lymphocyte ratio (NLR) was different among all groups and was higher in children aged ≥4 years, as compared with other groups. Following IVIG, the relative neutrophil and lymphocyte counts of all children with KD returned to normal levels. The altered levels of neutrophils and lymphocytes were found to be linearly correlated. The correlation coefficient in the five groups was 0.99, 0.87, 0.91, 0.97 and 0.99, from young to old, respectively (*p* < 0.01). The age of children with KD was positively correlated with older age (*r* = 0.91, *p* = 0.03). In patients aged ≥4 years, the absolute CD19^+^ B cell count prior to IVIG increased, and that increase was linearly correlated with the decrease in interleukin-10 (IL-10) following IVIG (*r* = 0.71, *p* < 0.05). The older the child's age, the better the regulatory effect of IVIG on the KD child's immune response and the recovery of immune equilibrium it achieved. In KD patients aged ≥4 years, the abnormally proliferating CD19^+^ B cells may be involved in the secretion of IL-10 to balance the humoral immunity. In such patients, the combination of the absolute CD19^+^ B cell count prior to IVIG and the decreased levels of IL-10 following IVIG may play a crucial role in evaluating the effect of IVIG in the inflammation.

## 1. Introduction

Mucocutaneous lymph node syndrome, also known as Kawasaki disease (KD), is an acute, self-limiting systemic inflammation that commonly occurs as vasculitis in pediatric diseases. The serious complications of KD are the leading cause of acquired heart diseases among children, such as coronary artery lesions (CALs), including dilations and aneurysms [1]. During the acute period of this disease, immunoglobulin intravenous (IVIG), when used timely, is the predominant therapeutic regimen for CALs, with a well-established efficacy in depressing their development [2]. However, at present, only the amelioration of clinical symptoms such as fever, rash, and conjunctivitis can be used to evaluate the therapeutic effect of IVIG. During this course of treatment, the influence of IVIG on the laboratory inflammatory response parameters of children with KD is not very clear. The identification of a treatment to follow IVIG is a therapeutic challenge. Identifying sensitive and suggestive inflammatory parameters can be helpful in developing a subsequent individualized therapy for children with KD.

As children grow up, the composition and the maturity of their immune system varies by age and circumstance [3]. Studies have shown that the lymphocyte percentage gradually decreases, and the neutrophil percentage increases annually, as children grow up [4, 5]. It is therefore understandable that the inflammatory response differs among children with KD from diverse age groups.

Neutrophils play a dominant role in the early and acute phase of KD through their increased count in the peripheral blood and the infiltration at the necrotizing arteritis [6, 7]. Furthermore, it has been reported that the neutrophil/lymphocyte ratio (NRL) can be used to evaluate inflammation in patients with KD [8, 9]. It would therefore be useful to investigate the distribution and association between these two subgroups of blood cells involved in the immune response of KD children from different age groups when trying to gain insights into the therapeutic effect of IVIG.

Previous reports have revealed that, during the acute phase of KD, the plasma level of interleukin-10 (IL-10) in KD patients was clearly higher than that in patients at the convalescent phase and that of the control children [10]. Other studies found that the elevated level of IL-10 during the acute phase of KD decreased immediately after IVIG administration, coinciding with a rapid improvement in inflammatory symptoms. The mRNA IL-10 expression was detected in both human T and B cells [7, 11]. Further studies have revealed that the IL-10 genetic polymorphisms exert an important effect on CALs in acute KD and that ATA genotyping is significantly associated with an increased risk of CALs [12]. It was recently found that IL-10, as an immune-regulatory cytokine, functions as a switch for lymph proliferation in human T cell leukemia-related diseases, in addition to the suppressive effect on the Th1 response and inflammation [13]. The present study also explored the role of IL-10 and its possible association with certain subgroups of lymphocytes during treatment for acute-phase KD.

Focusing on the inflammatory response in KD children from different age groups before and after IVIG, the aim of the present study was to explore some suggestive laboratory parameters and delineate the role of neutrophil and lymphocyte subgroups together with cytokines in inflammatory change, in order to develop individualized treatment to follow IVIG.

## 2. Materials and Methods

### 2.1. Aim, Design, and Setting of the Study

This study is retrospective, and all the research was approved by the ethics committee of the Xinhua Hospital affiliated to Shanghai Jiao Tong University School of Medicine which lies in Shanghai, China. This study was exempt from informed consent, as the patients' general information, medical history, and laboratory data were obtained from past medical records and a database. The detection of lymphocyte subgroups using FACS analysis or cytokine measurement was performed in accordance with the clinical guides included in the whole therapeutic regiment, and the parents of children with KD provided their written informed consent upon the children's hospitalization. Exemption from informed consent was granted for this study (approval no. XHEC-D-2019-088). This retrospective study was conducted in the Xinhua Hospital. The data that support the present findings are available from the Xinhua Hospital, but not publicly. Restrictions apply to the availability of these data; a license of use was obtained for the present study. Data can also be made available from the authors upon reasonable request and with the permission of the Xinhua Hospital.

Patients' charts and electronic medical records were retrospectively reviewed. Laboratory parameters were collected from a customized database and included age, gender, and relative or absolute counts of neutrophils, lymphocytes, monocytes, and platelets from 1 day before and 2 days after IVIG treatment. The expression level of inflammatory factors, including tumor necrosis factor (TNF), IL-1, IL-2 receptor, and IL-6, from 1 day before and 3 days after IVIG treatment were also recorded. Data on lymphocyte subgroups from all children with KD were obtained from their medical records.

### 2.2. Patients

A total of *5*0 children diagnosed with KD between April and December in 2018 at the Xinhua Hospital affiliated to Shanghai Jiao Tong University School of Medicine were enrolled in this retrospective study. All diagnoses were performed based on consistent diagnostic criteria and were in accordance with the protocol outlined in “Diagnosis, Treatment and Long-Term Management of Kawasaki Disease” by the American Heart Association [7]. Classic KD was diagnosed in the presence of fever for ≥5 days, when combined with a minimum of 4/5 of the following principal clinical features: (i) bilateral bulbar conjunctival injection without exudate; (ii) erythema and cracking of lips, oral cavity, and/or pharyngeal mucosa; (iii) erythema and edema in the peripheral extremities; (iv) polymorphous skin rash; and (v) nonpurulent cervical lymphadenopathy. Patients with the following findings were excluded from the study: (i) incomplete collection of clinical and laboratory data; (ii) serious cardiovascular, hepatic or renal diseases, and primary disease associated with tumors, hematological diseases, congenital malformations, genetic metabolic diseases, primary myocarditis, or other primary diseases of major organs; and (iii) relapse that required retreatment.

In the present study, children with KD were from different age intervals and therefore at different stages of development and maturity. In order to perform the analysis accurately, the 50 children were divided into five groups according to their age (from younger to older). Patients were grouped as follows: (i) <1 year (<12 months), (ii) 1 year old (≥12 months but <24 months), (iii) 2 years old (≥24 months but <36 months), (iv) 3 years old (≥36 months but <48 months), and (v) ≥4 years (≥48 months).

### 2.3. Procedures and Samples

According to the previously published statement and protocol [7], once diagnosed with KD, a child received IVIG therapy (single dose of 2 g/kg) within 24 h. One day before and 3 days after IVIG treatment, all children underwent a peripheral blood leukocyte differential count test, using a full automatic hematology analyzer. Inflammatory cytokines were also detected, using a solid-phase enzyme labeled chemiluminescentimmunometric assay (Siemens AG). One day before IVIG treatment, lymphocyte subgroup counts were detected using flow cytometry (BD CANTO Plus, BD Biosciences).

### 2.4. Statistical Analysis

Statistical analysis was performed using SPSS 22.0 (IBM Corp., GraphPad Prism 6.0 (GraphPad Software, Inc.) and Excel 2010 (Microsoft Corporation) software. Clinical data are presented as the median (range), mean ± standarddeviation, number (*n*), or percentage (%). The distribution of males and females in each group was compared with one another by paired *t*-test. One-way ANOVA or paired *t*-test was also used to compare the corresponding normal values published in the literature with the mean, upper, and lower limits of the neutrophil percentage prior to IVIG treatment in children from different age groups [4]. The lymphocyte percentage was also measured in each group. The percentage of decreased neutrophils and that of increased lymphocytes was calculated based on the white blood cell differential count before and after IVIG, and the correlation between them was analyzed using SPSS 22.0 and GraphPad Prism 6.0 software. Next, correlation coefficients were obtained. In the ≥4 year group, the concentration of inflammatory factors before and after IVIG treatment was determined, and the degree of change of each inflammatory factor following IVIG treatment was calculated. The correlation between the change magnitude of inflammatory factors and the absolute count of different lymphocytes, including that of CD19^+^ B cells, prior to IVIG treatment was analyzed. Hypothesis test of *r* using *t*-test was performed to test whether the coefficient of product-moment correlation (*r*) from the sample is suitable for the population. A two-tailed *p* < 0.05 was considered to be statistically significant.

## 3. Results

### 3.1. Characteristics of Children with KD

**Figure 1:**
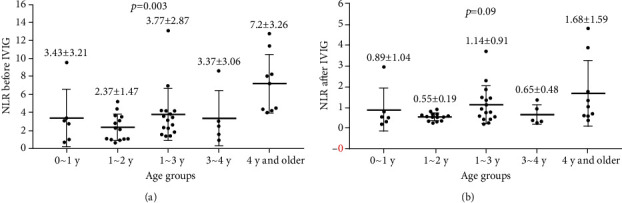
(a, b) NLR before and after IVIG in different age groups. (a) NLRs prior to IVIG in children with KD were clearly different among different age groups. (b) Following IVIG, NLRs were not significantly different among different age groups. NLR: neutrophil/lymphocyte ratio; IVIG: immunoglobulin intravenous.

Table 1 shows the characteristics of 50 children with KD, including 27 boys (54%) and 23 girls (46%). The children were divided into five groups according to age. The numbers of children in each group were 6, 14, 16, 5, and 9, respectively. No differences in indexes, including gender distribution, therapeutic regiment with a high dosage of aspirin (including dosage and therapeutic duration) and immunoglobulin, extent of changes in neutrophil and lymphocyte percentage, decrease in level of IL-10, relative CD19^+^ B cell count, and NLR following IVIG, were observed among the groups. There were clear differences in age, body weight, and absolute CD19^+^ B cell count. An obvious difference in the mean NLR prior to IVIG was observed among the five groups (*p* < 0.03). In the ≥4 year group, the mean NLR prior to IVIG was 7.2 ± 3.26, which was higher than that in other groups. Following IVIG treatment, NLR returned to normal, and no significant difference was observed among the five groups (Figure 1).

### 3.2. All Acute-Phase KD Patients Undergo Neutrophilia and Lymphocytopenia and Then following IVIG Treatment, with a Decrease in the Neutrophil and an Increase in the Lymphocyte Percentage in all Groups

As shown in Table 2, the relative neutrophil and lymphocyte counts of children with KD exhibited obvious differences, when compared with the normal values and ranges newly published, which are based on healthy children from different age groups [4]. The relative counts of neutrophils were clearly increased, while those of lymphocytes were clearly decreased. Following IVIG, both indexes returned to normal. No differences were observed, as compared with the normal values (Table 3). Since the relevant normal values for infants aged <1 year could not be found, only data from children aged ≥1 year were analyzed.

### 3.3. Correlation Coefficient between the Decreased Neutrophil and Increased Lymphocyte Count Increases with Older Age

**Figure 2:**
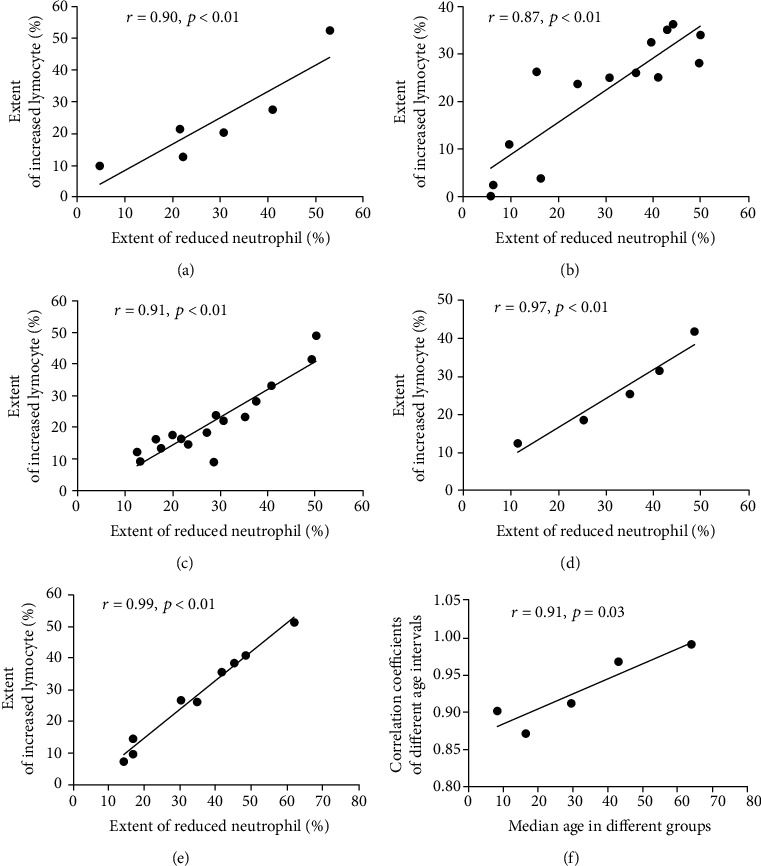
The reduced neutrophil percentages were positively correlated with the increased lymphocyte percentages following treatment with IVIG in all groups. Grouped by age from young to old, the Spearman correlation coefficient (*r*) between the reduced neutrophil percentages and the increased lymphocyte percentages following IVIG was 0.90 (*n* = 6), 0.87 (*n* = 14), 0.91 (*n* = 16), 0.97 (*n* = 5), and 0.99 (*n* = 9), respectively. Children were divided into five groups based on age: (a) <1, (b) 1-2, (c) 2-3, (d) 3-4, and (e) ≥4 years. (f) Positive correlation between the coefficients from the changes in neutrophils and lymphocytes and the median ages of children in all groups. *r* = 0.91, *p* = 0.03. IVIG: immunoglobulin intravenous.

As shown in Figure 2(a)–2(e), following IVIG, the extent of the decrease in the neutrophil percentage was positively correlated with the extent of the increase in the lymphocyte percentage in all five groups. The correlation coefficient in the five groups (from younger to older) were 0.90 (*n* = 6, *p* = 0.01), 0.87 (*n* = 14, *p* < 0.01), 0.91 (*n* = 16, *p* < 0.01), 0.97 (*n* = 5, *p* = 0.0065), and 0.99 (*n* = 9, *p* < 0.01; *p* < 0.01 in all groups). This type of correlation was not found between any other two cell subgroups or ingredients in the peripheral blood (*r* = 0.91, *p* = 0.03; Figure 2(f)).

### 3.4. In KD Children Aged ≥4 Years, the Absolute CD19^+^ B Cell Count prior to IVIG Is Correlated with the Decreased Level of IL-10 following IVIG

In the ≥4 year group (*n* = 9), the mean value for the absolute CD19^+^ cell count was 495.11 cells/*μ*l and the standard deviation was 250.5 cells/*μ*l prior to IVIG, which were significantly higher than the normal standardized threshold established in our hospital. The normal mean value for the absolute CD19^+^ cell count is 224.5 cells/*μ*l, and the corresponding standard deviation is 152.03 cells/*μ*l for children aged ≥4 years (*n* ≥ 200).

**Figure 3:**
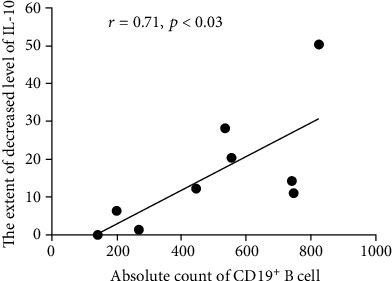
In the group of KD children aged ≥4 years, the absolute CD19^+^ B cell counts prior to IVIG treatment were positively correlated with the decreased expression level of IL-10 following IVIG treatment. *r* = 0.91; *p* = 0.03. KD: Kawasaki disease; IVIG: immunoglobulin intravenous; IL-10: interleukin-10.

At the same time, in this group, the mean expression level of IL-10 prior to IVIG was as high as 21.34 pg/ml, clearly higher than the normal value (<5 pg/ml). Following IVIG, the mean level of IL-10 in was significantly reduced to 5.3 pg/ml. The absolute CD19^+^ cell count prior to IVIG treatment was correlated with the decreased level of IL-10 following IVIG treatment (Figure 3). The correlation coefficient was 0.71 with a *p* = 0.03. After the hypothesis test of the *r*, the *t*_*r*_ was 2.68 with the two-tailed *p* < 0.05. No correlation was identified between other inflammatory cytokines, including IL-1, TNF-*α*, IL-2 receptor, and IL-6, and other lymphocyte subgroups, such as CD4^+^, CD8^+^ or CD16^+^56^+^ cells.

## 4. Discussion

A total of 50 children with KD were included in this retrospective study and divided into five groups, according to age. There were no obvious differences in gender distribution in terms of the incidence of KD in any of the groups. Other studies have reported that the disease occurs more frequently in boys [14, 15]. The present results were inconsistent with the results of those studies, but consistent with the data recently reported from Australia [16]. A study with a larger sample size will lead to more convincing conclusions.

Neutrophil recruitment is an early event induced by bacterial infections, excluding severe trauma, massive hemorrhage, malignancy, and chemical poisoning [17, 18], with lymphocyte loss usually considered an early biomarker of the systemic spread of severe infection [19]. The present results revealed that, in the acute phase of KD, abnormally elevated neutrophil percentages and decreased lymphocyte percentages prior to IVIG are the main laboratory characteristics of children with KD. Studies have reported that the NLR could serve as a rapid and simple parameter and severity index of inflammation that reflects the intensity of stimulation and inflammatory response. Physiologically, the NLR is <5.0. Under pathologic conditions and in severe inflammation, the NLR is increased to >6 [20]. An investigator from Japan suggested that the use of the combination of NLR ≥ 3.83 and platelet to lymphocyte ratio > 150 could sensitively and specifically predict IVIG resistance or CAL development in children with KD [8]. In the present study, the highest NLR value was identified in children from the ≥4 year group, suggesting that a more intense inflammatory response may occur in those children. However, whether there is a higher risk of CAL development or IVIG resistance needs to be treated with caution. In fact, in the present study, the high NLR that was observed prior to IVIG in the ≥4 year group returned to normal rapidly after IVIG, and no difference was observed among groups. This phenomenon suggested that children with KD aged ≥4 years respond rapidly to IVIG treatment and that IVIG plays a more critical role in the immune regulation of these older children with KD than in that of younger children. PLR was not studied in the present study, suggesting that the children with KD enrolled in this study may have different characteristics from those enrolled in previous studies, and they were not the same type [8, 21].

Based on the above results, it was further found that, along with the increase in the age of children with KD, the correlation coefficient (*r*) between the decreased neutrophil percentage and the increased lymphocyte percentage increased. It is well known that in children younger than 2 years old, adaptive immunity does not really approach that of healthy adults, and full immune competence is not truly reached until teenage [22]. Studies showed that the percentage of lymphocytes gradually decreased and that of neutrophils increased annually among children aged 1-7 years. During this period, the percentage medians and ranges of lymphocytes and neutrophils changed as children from different age groups grew [4, 5]. Thus, children with KD may have different inflammatory responses varied with age.

Based on the results shown in Tables 1–3 and Figure 1, the result shown in Figure 2 suggested that IVIG has a better immune-modulatory effect on older (≥4 years) than that in younger KD children and could contribute more rapidly to gain balance between cellular subgroups, including subgroups of neutrophils and lymphocytes. This type of effect was found to increase with the increase in the age of children with KD.

The results of the present study suggested that KD children aged ≥4 years may benefit more from IVIG than younger children with KD. The age of ≥4 years may be a favorable factor in the treatment of children with KD during the course of IVIG, contributing to the rapid recovery and balance of KD children's immune response.

CD19^+^ B cells are immunoglobulin-producing cells and play a major role in the humoral immune response. In the present study, during the acute phase of KD in children, the clearly increased absolute CD19^+^ B cell count prior to IVIG was evident of its activation and proliferation under the stimulation of antigens and its predominant role in the patients' activated humoral immunity. It has been reported that, in mice, CD19 enhances B cell receptor- (BCR-) induced signaling, which is crucial for the activation and proliferation of B cells and the subsequent enhanced response of humoral immunity [23, 24]. This finding provided evidence that proliferating CD19^+^ B cells play a dominant role in the activation of humoral immunity of KD children aged ≥4 years during the acute phase of the disease.

Furthermore, in the present study, the linear correlation between the decreased level of IL-10 and the absolute CD19^+^cell count suggested that CD19^+^ B cells may play a role in IL-10 secretion. A previous study found that various types of cells, including Th2 cells, macrophages, DCs, mast cells and even neutrophils, can secret IL-10 [25]. Another study confirmed that B cells, when in deficiency of CD19, could not produce a higher IL-10 than that of wild-type B cells. In a CD19^−/−^ mouse model, the animals displayed a reduced production of IL-10 by B cells and developed a more serious disease [26]. It has also been reported that B cells from patients with multiple sclerosis secreted markedly lower levels of IL-10, as compared with healthy donors [27], suggesting the possible IL-10 secretion function of B cells in humans. Combined with the present results, it is reasonable to speculate that an abnormally high CD19^+^ B cell count may be the result of an overactive humoral immunity in KD patients. These proliferating CD19^+^ B cells can secret IL-10, as a compensatory response in an attempt to keep maintain immune response balance.

Following IVIG treatment, the patients' IL-10 levels were significantly reduced, suggesting that the absolute CD19^+^ B cell count had a tendency to decrease. Considering another phenomenon that the patients' mean lymphocyte percentage increased while the relative neutrophil count decreased following IVIG, we speculated that other lymphocyte subgroups, such as T cells, which act as antigen-presenting or cytokine-producing cells, may also be activated to proliferate. As the functions of immunoglobulin are complex [28], IVIG could bind to the siglecs expressed on the surface of neutrophils and result in cell death [29]. In addition, IVIG containing Fc has the ability to stimulate the expression of a population of natural regulatory T cells [30]. Therefore, we speculated that, following IVIG treatment and along with the inhibition of both the neutrophil count and the excessively activated humoral immunity, the body's cellular immunity function would recover and the other major ingredients of T cells would proliferate contributing to the boost of cellular immunity in KD children aged ≥4 years, whose immune system was approaching maturation. In children with KD from this age group, the combination of absolute CD19^+^ B cell count prior to IVIG and the decreased level of IL-10 following IVIG can be used as a potential sensitive indicator of the body's inflammatory response status and an effective evaluator of IVIG treatment.

To the best of our knowledge, the present study was the first to present data from KD children aged ≥4 years showing that CD19^+^ B cells are stimulated to be activated and proliferate, leading to the enhanced function of humoral immune response. Based on the results we speculate that, in children with KD, activated proliferating CD19^+^ B cells can secret IL-10, and IVIG can inhibit this secretion, thereby inhibiting cellular immunity, activating humoral immunity, and regulating immune response. However, this needs to be confirmed in more laboratory data and cases.

This retrospective analysis was not without its limitations. The first limitation was the small number of cases enrolled, and the fact that the data was collected from a single hospital and therefore cannot accurately reflect the overall epidemiology of KD.

The second limitation was that, before and after IVIG treatment, the absolute cell count of lymphocyte subgroups, particularly that of CD19^+^ B cells, should be monitored dynamically in order to observe the trend of lymphocyte subsets more clearly.

Another limitation was that it was impossible to verify the effects following the elimination of CD19^+^ B cell function, which cannot occur in patients. However, this could be tried in mice. It is also possible to collect a subset of cells and culture them *in vitro* to study the IL-10 secretion function of CD19^+^ B cells.

In our next study, we will enroll more KD children aged ≥4 years. During the treatment course of children with KD, more detailed examinations will be performed to study in-depth and more parameters will be monitored dynamically in order to clearly demonstrate the mechanism underlying the association between the CD19^+^ B cell count and the expression level of IL-10.

## 5. Conclusions

In conclusion, IVIG has a better immune-modulatory effect on older than on younger children with KD. The age of ≥4 years may be a favorable factor in the IVIG treatment of children with KD. During the therapeutic period of IVIG, CD19^+^ B cells were stimulated to proliferate abnormally and were found to play a dominant role in the activated humoral immunity of KD children aged ≥4 years. The linear correlation between the absolute cell count of CD19^+^ prior to IVIG and the decreased level of IL-10 following IVIG suggested the possibility of IL-10 secretion by CD19^+^ B cells in KD children aged ≥4 years. The combination of these two indexes could serve as a marker for evaluating the sensitivity of the inflammatory response and therapeutic effect of IVIG on children with KD aged ≥4 years.

## Figures and Tables

**Table 1:** Clinical and biochemical characteristics of the Kawasaki disease children.

Characteristics	Total	0-1 year	1-2 years	2-3 year	3-4 years	Above 4 years	*p* value
Patients (*n*)	50	6	14	16	5	9	—
Gender (m)(*n* (%))	27 (54)	4 (66.7)	9 (64.3)	8 (50)	2 (40)	4 (44.44)	0.77
Age (months)	28 (16-42.25)	8 (3.00-11.00)	16.5 (15-19.25)	29.5 (26.50-31)	43 (41.5-45)	64 (58.5-75.5)	<0.01
Body weight (kg)	13.38 (10.75-16.63)	7.8 (7.37-9.13)	11.03 (10-12)	14 (13-15)	15 (14.4-16.6)	18 (17.75-20.75)	<0.01
Aspirin dosage (mg/kg.dose)	11.11 (10.00-12.50)	10.27 (8.39-11.42)	12.5 (10-13.59)	11.22 (10.09-12.41)	11.63 (8.33-13.50)	11.11 (9.76-11.27)	0.38
Aspirin frequency (doses/day)	3	3	3	3	3	3	0.23
Duration of high dose aspirin (days)	4 (4-5)	4 (3.75-6.75)	4 (4-5)	5 (3.25-5.75)	3 (2.5-5.0)	4.0 (3.5-4.5)	0.61
IgG dosing (g/kg)	1.96 (1.90-2.00)	1.97 (1.84-2.03)	1.95 (1.86-2.05)	1.96 (1.92-2)	2 (2-2.02)	1.94 (1.49-1.97)	0.37
The reduced extent of neutrophil percentage (%)	30.22 ± 14.39	28.87 ± 16.82	29.52 ± 16.11	28.21 ± 11.76	34.42 ± 14.48	34.58 ± 16.48	0.87
The increased extent of lymphocyte percentage (%)	23.70 ± 4.44	24.00 ± 15.31	22.14 ± 12.48	21.86 ± 11.31	25.98 ± 11.37	27.92 ± 15.00	0.76
The decreased IL-10 (pg/ml)	15.05 (6.32-52.33)	10.7 (8.18-49.38)	42.65 (14.98-75.18)	11.85 (2.49-31.78)	14.5 (0.7-63.32)	12.2 (3.83-24.35)	0.35
The absolute count of CD19^+^ B cell(cells/*μ*l)	857.2 (520.44-1285.46)	1043.83 (419.02-1736.44)	1176.9 (1019.37-1954.14)	579.29 (432.90-997.5)	1220.95 (918.06-1322.56)	534.64 (233.96-743.67)	<0.01
CD19^+^ B cell (%)	29.20 ± 9.09	27.41 ± 8.03	32.64 ± 9.55	26.76 ± 10.62	33.30 ± 5.74	27.08 ± 6.28	0.305
NLR before IVIG	3.91 ± 3.06	3.43 ± 3.21	2.37 ± 1.47	3.77 ± 2.87	3.37 ± 3.06	7.2 ± 3.26	<0.01
NLR after IVIG	0.99 ± 0.98	0.89 ± 1.04	0.55 ± 0.19	1.14 ± 0.91	0.65 ± 0.48	1.68 ± 1.59	0.077

Values are expressed as mean ± standarddeviation, number, or median (*Q*_1_-*Q*_3_). NLR: neutrophil lymphocyte ratio.

**Table 2:** Patients' means of neutrophil percentage and lymphocyte percentage before the treatment of IVIG in different age groups.

	<1 y (*n* = 6)	1 y (*n* = 14)	2 y (*n* = 16)	3 y (*n* = 5)	≥4 y (*n* = 9)
Mean of neutrophil (%) (upper limit, lower limit)	60.18 (80.98, 39.39)	57.92^∗∗^ (66.45, 49.4)	67.68^∗∗^ (73.66, 61.71)	62.36^∗∗^ (81.75, 42.98)	80.22^∗∗^ (83.68, 76.76)
Mean of lymphocyte (%) (upper limit, lower limit)	26.9 (41.6, 12.2)	31.34^∗∗^ (38.6, 24.07)	23.02^∗∗^ (27.45, 18.59)	26.58^∗∗^ (41.64, 11.52)	13.02^∗∗^ (9.34, 16.71)

^∗∗^Compared with the normal value established in newly published literature [4], *p* < 0.01.

**Table 3:** Patients' characteristics and the means of neutrophil percentage and of lymphocyte percentage after the treatment of IVIG in different age groups.

	<1 y (*n* = 6)	1 y (*n* = 14)	2 y (*n* = 16)	3 y (*n* = 5)	≥4 y (*n* = 9)
Mean of neutrophil (%) (upper limit, lower limit)	31.32 (50.10, 12.53)	28.4 (32.63, 24.17)	39.48 (47.71, 31.24)	29.94 (47.68, 12.20)	45.64 (57.80, 33.49)
Mean of lymphocyte (%) (upper limit, lower limit)	50.9 (69.02, 32.78)	53.47 (57.74, 49.20)	44.88 (52.99, 36.76)	52.56 (67.49, 37.63)	40.94 (54.19, 27.70)

## Data Availability

The data that supports the findings of this study are available from Xinhua Hospital but restrictions apply to the availability of these data, which is used under license for the current study, and so are not publicly available. Data is however available from the authors upon reasonable request and with the permission of Xinhua Hospital.
